# Draft Genome Sequence of Mercury-Resistant *Serratia* sp. Strain SRS-8-S-2018

**DOI:** 10.1128/MRA.00136-20

**Published:** 2020-04-09

**Authors:** Sherif Gendy, Ashish Pathak, Meenakshi Agarwal, Rajesh Singh Rathore, Ashvini Chauhan

**Affiliations:** a Environmental Biotechnology Laboratory, School of the Environment, Florida A&M University, Tallahassee, Florida, USA; b School of Allied Health Sciences, Florida A&M University, Tallahassee, Florida, USA; University of Southern California

## Abstract

A mercury (Hg)-resistant *Serratia* sp. strain, SRS-8-S-2018, was isolated, followed by generation of its draft genome sequence, which indicated a genomic size of 5,323,630 bp composed of 5,261 coding sequences. A suite of genomic functions in strain SRS-8-S-2018 was identified, and these likely facilitate survival in a metalliferous soil habitat.

## ANNOUNCEMENT

The Savannah River Site (SRS) is located on the northeast bank of the Savannah River (South Carolina) and consists of some areas that are historically contaminated by former nuclear material production activities (1–3). Mercury is one such contaminant, but microbial communities under long-term contaminant exposure can develop mechanisms to resist and biotransform Hg. To better understand the genomic underpinnings of microbial Hg resistance, SRS soils were plated onto lysogeny broth (LB) agar supplemented with 5 μg/ml of mercuric chloride and incubated at 30°C; colonies were isolated as reported before (4). One Hg-resistant strain, called SRS-8-S-2018, was obtained and identified as a *Serratia* sp. strain using Sanger sequencing of the 16S rRNA gene on DNA extracted using the ZR fungal/bacterial DNA kit (Zymo Research, Irvine, CA, USA), amplified with primers 27F (AGAGTTTGATCMTGGCTCAG) and 1429R (TACGGYTACCTTGTTACGACTT) using DreamTaq green PCR master mix (Thermo Fisher). The PCR conditions were 95°C for 5 min followed by 30 cycles of 95°C for 30 s, 57°C for 45 s, and 72°C for 2 min. After cleanup with ExoSAP-ITPCR cleanup reagent (Thermo Fisher), amplicons were sequenced on an ABI 3730xl capillary sequencer, employing 27F as the sequencing primer. The obtained 16S rRNA gene sequence was analyzed using NCBI BLAST (5).

MIC analysis was conducted as shown before (6) and revealed the strain’s Hg resistance up to 10 μg/ml Hg (https://doi.org/10.6084/m9.figshare.12008832.v1). The draft genome sequence of strain SRS-8-S-2018 was obtained using DNA extracted from cells grown overnight in LB liquid medium following the Nextera XT kit instructions (Illumina, San Diego, CA) and was sequenced with an Illumina NextSeq 500 instrument, employing paired-end 2 × 75-base reads. A total of 4,981,764 clusters were generated (9,963,528 reads total), and raw reads were trimmed using CLC Genomics Workbench v11 (Qiagen), employing a Q20 trimming threshold and no tolerance for degenerate bases. *De novo* assembly was performed using no-scaffold default settings within the CLC Genomics Workbench v11.0.1 and a minimum length of 200 bases for contigs. Reads were mapped against the assembled scaffolds within CLC Genomics Workbench, employing default settings (mismatch cost = 2, insertion cost = 3, deletion cost = 3, length fraction = 0.5, similarity fraction = 0.8). Coverage statistics were computed using this mapping, and contigs with low average coverage (<10) were removed. All bioinformatics-based analysis was conducted using default parameters unless otherwise specified.

The genome of SRS-8-S-2018 had a coverage of 106× and was assembled over 159 contigs with an *N*_50_ value of 101,301 bases, an *L*_50_ value of 17, and a size of 5,323,630 bp with an average GC content of 59.48%. Genome annotation was performed using the NCBI Prokaryotic Genome Automatic Annotation Pipeline (PGAAP) (7) and Rapid Annotations using Subsystems Technology (RAST) (8), which revealed a coding sequence of 5,261 proteins (CDS) and 55 genes for tRNA. A total of 515 subsystems were identified, with genes related to biodegradation, stress response, and membrane transport and several gene determinants for resistance against heavy metals, including the mercury-regulatory MerR, which likely facilitates the survival of strain SRS-8-S-2018 in an Hg-contaminated habitat.

### Data availability.

The whole-genome shotgun project of *Serratia* sp. SRS-8-S-2018 reported in this study has been deposited at DDBJ/ENA/GenBank under the accession number VHNF00000000, BioProject number PRJNA550097, and BioSample number SAMN12107717. The version described in this research is version VHNF01000000. Raw sequence data for strain SRS-8-S-2018 are available under SRA run accession number SRR11284024.
